# The presence of a draining sinus is associated with failure of re-implantation during two-stage exchange arthroplasty

**DOI:** 10.5194/jbji-7-55-2022

**Published:** 2022-03-22

**Authors:** Alexandra S.​​​​​​​ Gabrielli, Alan E. Wilson, Richard A. Wawrose, Malcolm Dombrowski, Michael J. O'Malley, Brian A. Klatt

**Affiliations:** Department of Orthopaedic Surgery, University of Pittsburgh Medical Center, Pittsburgh, PA 15232, United States of America

## Abstract

**Background**: Reinfection rates after two-stage exchange arthroplasty for prosthetic joint infection (PJI) have been reported as high as 33 % in the literature. Understanding risk factors for treatment failure will help to preoperatively counsel patients on the likelihood of successful treatment and possibly influence the surgeon's treatment algorithm. This study aimed to delineate whether the presence of a draining sinus tract is associated with risk of failure of two-stage exchange arthroplasty. **Methods**: We performed a single institution, multi-center retrospective chart review of outcomes of patients treated for PJI with two-stage exchange arthroplasty between June 2006 and May 2016. For patients treated prior to 2011, PJI was defined based on the preoperative work-up and intraoperative findings as determined by the attending surgeon. After 2011, PJI was defined using MSIS consensus criteria. All patients had a minimum of follow-up of 2 years or treatment failure prior to 2 years. Treatment failure was defined as reinfection or failure to complete two-stage exchange secondary to persistent infection or other host factors. Operative reports and clinical notes were reviewed to assess for presence of a draining sinus tract. **Results**: 240 patients were treated for PJI with intended two-stage exchange arthroplasty. The overall rate of treatment failure was 29.6 % ( 71/240 ), while the overall rate of reinfection was 13.3 % ( 32/240 ). A total of 39 patients did not complete second stage revision; final treatment for these patients was amputation, fusion, or chronic antibiotic suppression. A total of 52 of 240 patients (21.7 %) had a draining sinus tract at presentation. Patients with a sinus tract were significantly *less* likely to be replanted compared to those without a sinus tract at presentation (13.3 % vs. 26.9 %, p=0.02 ). However, when accounting for all mechanisms of treatment failure, including reinfection following replantation, there was no statistically significant difference detected between the sinus and no-sinus groups (27.7 % vs. 36.5 %, p=0.22 ). **Discussion**: A draining sinus tract represents a chronic, deep infectious process with ultimate compromise of overlying soft tissues. Thus we hypothesized it would be associated with failure in a two-stage exchange arthroplasty. These data demonstrate that patients with a draining sinus are significantly less likely to undergo re-implantation. This provides evidence to the paucity of data surrounding draining sinuses and two-stage PJI treatment.

## 1 Introduction

Joint arthroplasty remains one of the most successful surgeries in modern medicine with many series demonstrating high patient satisfaction and greater than a 95 % survivorship at 10-year follow-up (Furnes et al., 2001; Older, 2002). Unfortunately, between 0.6 % and 2.2 % (Dale et al., 2012; Ong et al., 2009) of patients who have undergone joint arthroplasty develop a postoperative prosthetic joint infection (PJI), which can lead to devastating consequences including reduced quality of life and even death (Zmistowski et al., 2013). The continued global surge in the number of total joint arthroplasties performed annually is predicted to result in a subsequent increase of PJI (Kurtz et al., 2007), making it paramount that surgeons understand how to best manage this dreaded complication.

There exists a multitude of therapeutic management options available for treating PJI, and the chosen strategy is generally based upon chronicity of infection, pathogen, as well as host factors. In acute PJI with fewer virulent pathogens, single-stage treatment with surgical debridement, antibiotic treatment, and implant retention (DAIR) has been employed with better functional results than a two-stage revision (Byren et al., 2009). However, in patients with chronic PJI or with particularly infectious pathogens, two-stage treatment consisting of initial explanation of components and later re-implantation of components may be required. Approximately half of patients diagnosed with PJI will undergo major revision surgery (Lindgren et al., 2014), either in a single-stage or in a two-stage fashion. Of these two revision strategies, a two-stage exchange approach of revision arthroplasty for PJI remains standard for many surgeons (Cooper and Della Valle, 2013).

Although many studies have reported infection eradication in up to 90 % of cases following two-stage revision surgery, these studies often fail to consider the attrition of patients that occurs in the inter-stage period between explant and re-implantation (Chen et al., 2014; Gomez et al., 2015; Parvizi et al., 2011). When accounting for all failures of two-stage revision surgery, the rate of failure has been reported to be as high as 40 % (Ford et al., 2018; Wang et al., 2019). Unfortunately, there exist a paucity of data regarding predictive factors for failure of these two-stage revisions, as well as a recognized criterion for patients to meet to consider re-implantation. This can make it challenging for surgeons to properly counsel their patients. The aim of this study was to discern whether the presence of a draining sinus prior to two-stage revision is associated with risk of failure of re-implantation, as well as to compare the rate of overall treatment failure to those without a draining sinus. Because the presence of a draining sinus represents a chronic infectious process that ultimately compromises the overlying soft tissues, it was hypothesized to be a risk factor for failure of re-implantation and for overall failure of two-stage exchange arthroplasty.

## 2 Methods

This retrospective chart review was approved by the Institutional Review Board, and all patients that were treated for PJI with two-stage exchange arthroplasty at one hospital within our hospital system between June 2006 and May 2016 were evaluated. For patients treated prior to 2011, PJI was defined based on the preoperative work-up and intraoperative findings as determined by the attending surgeon. For patients treated after 2011, PJI was defined using MSIS consensus criteria (Parvizi and Gehrke, 2014).

Patients without either (1) a minimum follow-up of 2 years or (2) treatment failure prior to 2 years were excluded from this study (Xu et al., 2020). Treatment failure was defined as failure to complete the two-stage exchange or reinfection after re-implantation. Reinfection was defined by MSIS criteria. The remaining patients were divided into two cohorts based on the presence or absence of a draining sinus at their presentation. The presence of a draining sinus presence was determined by review of clinical and operative notes in the electronic medical record.

For each patient, demographic data (including age, BMI, gender, and smoking status) were recorded. Additionally, site of arthroplasty (hip or knee), microbiology data, and MSIS Host Grade were also recorded. Patient host grade was determined based upon MSIS Host Grade criteria (McPherson et al., 2002). For each cohort (sinus and no sinus), clinical course was reviewed and analyzed. Furthermore, for those patients who did not undergo re-implantation, treatment course was classified as (1) being placed on chronic antibiotic suppression, (2) undergoing amputation, or (3) undergoing fusion. The criteria for re-implantation used for this study were as follows: (1) the patient had completed at least 6 weeks of antibiotics, (2) had normalized inflammatory lab values (WBC, ESR, CRP), and (3) had normalized nutrition labs (prealbumin, albumin, total protein). The primary study endpoint was failure to complete two-stage exchange arthroplasty. In addition, the rate of overall two-stage exchange arthroplasty was recorded and compared between cohorts, as defined above.

Chi-squared analysis was performed to analyze differences in gender, smoking status, site of arthroplasty, and MSIS host grade between the two groups. A Student t test was used to assess differences in age and BMI of the two groups. Chi-squared analysis was performed to identify differences in microbiology cultures between the two groups. In addition, the relative risk of failure to undergo re-implantation and overall treatment failure between the two cohorts was calculated. For all statistical tests, significance was set at p<0.05 .

## 3 Results

In total after exclusion criteria were applied, 240 patients were treated for PJI with the original intent of undergoing two-stage exchange arthroplasty (Table 1). Of these, 52 patients (21.7 %) had a draining sinus at presentation. Of the patients with a draining sinus, 35 were total knee arthroplasty (TKA) patients and 17 were total hip arthroplasty (THA) patients. In the non-sinus group, 135 were TKA patients and 53 were THA patients. Additionally, 46.1 % of the sinus tract group had an articulating spacer while 49.4 % of the non-sinus tract had an articulating spacer. Antibiotic content of the spacers was not documented consistently in the patient's chart.

Those presenting with and without a sinus tract at initial presentation shared similar demographics regarding age, sex, proportion of smokers, and the joint involved (all p>0.05 ). In addition, there were no significant differences noted between the host grades of the two cohorts ( p>0.05 ).

**Table 1:** Patient demographics. In total, 240 patients were treated for prosthetic joint infection of either the hip or the knee with intended two-stage exchange arthroplasty (all p>0.05 ).​​​​​​​

	No sinus tract	Sinus tract	P value
	( n=188 )	( n=52 )	
Demographics			
Average age (years)	62.0 ± 11.1	65.0 ± 11.3	0.09
BMI	34.7 ± 9.4	32.7 ± 7.1	0.16
Male	52.1 % ( 97/188 )	50 % ( 26/52 )	0.79
Smokers	19.6 % ( 37/188 )	23.1 % ( 12/52 )	0.58
Site of arthroplasty			
Knee	71.8 % ( 135/188 )	67.3 % ( 35/52 )	0.53
Hip	28.2 % ( 53/188 )	33.7 % ( 17/52 )	0.44
MSIS host grade			
A	25.5 % ( 48/188 )	21.2 % ( 11/52 )	0.52
B	50.5 % ( 95/188 )	59.6 % ( 31/52 )	0.25
C	23.9 % ( 45/188 )	19.2 % ( 10/52 )	0.48

The overall rate of treatment failure in the combined cohorts (sinus and no sinus) was 29.6 % ( 71/240 ). In addition, the overall rate of reinfection was 13.3 % ( 32/240 ), and the overall rate of failure to compete second stage revision was 16.3 % ( 39/240 ). In patients who did not complete second stage revision, 51 % ( 20/39 ) were treated with antibiotic suppression, 28 % ( 11/29 ) underwent fusion, and 21 % ( 8/39 ) underwent above the knee amputations.

It was noted that patients with a sinus tract were significantly less likely to be re-implanted compared to those without a sinus tract at presentation (13.3 % vs. 26.9 %, p=0.02 ) (Table 2). However, when accounting for all mechanisms of treatment failure, excluding failure to re-implant, there was no statistically significant difference detected between the sinus and no-sinus groups (27.7 % vs. 36.5 %, p=0.22 ). The relative risk of overall treatment failure given the presence of a draining sinus tract was 1.32 (95 % CI [0.86 to 2.02].

**Table 2:** Draining sinus as a risk factor. Patients with a draining sinus tract were significantly less likely to undergo re-implantation ( p=0.02 ).​​​​​​​

	No sinus tract	Sinus tract	P value
	( n=188 )	( n=52 )	
Failure of two-stage exchange			
Re-infection	14.4 % ( 27/188 )	9.6 % ( 5/52 )	0.37
Failure to re-implant	13.3 % ( 25/188 )	26.9 % ( 14/52 )	0.02
Chronic suppression	56 % ( 14/25 )	50 % ( 7/14 )	0.72
Fusion	28 % ( 7/25 )	14.3 % ( 2/14 )	0.34
Amputation	16 % ( 4/25 )	35.7 % ( 5/14 )	0.17
Total	27.7 % ( 52/188 )	36.5 % ( 19/52 )	0.22

Additionally, microbial data for each group are listed in Table 3. No organism was identified in culture in 35.1 % of the non-sinus tract and 30.7 % of the sinus tract patients. For patients with positive culture data, coagulase-negative staphylococcus was the most common organism identified in each of the groups. Systemic antibiotics were given under the care of our infectious disease team.

**Table 3:** Microbial culture data. No organism was identified in the majority of both the sinus tract and non-sinus tract cohorts.​​​​​​​

Culture organism	No sinus tract	Sinus tract
	( n=188 )	( n=52 )
No growth on culture	66 (35 %)	16 (31 %)
Coagulase-negative staphylococcus	30 (16 %)	11 (21 %)
MSSA	25 (13 %)	9 (17.3 %)
Viridans streptococci	7 (4 %)	1 (2 %)
MRSA	18 (10 %)	5 (10 %)
Poly-microbial	8 (4 %)	3 (6 %)
Group B or G *Streptococcus*	7 (4 %)	1 (2 %)
Other/not documented	27 (12 %)	6 (12 %)

Using chi-squared analysis, there was no statistically significant difference between the culture data of the two groups ( p=0.9438 ).

## 4 Discussion

Understanding risk factors for PJI treatment failure and failure to undergo re-implantation in two-stage exchange arthroplasty is necessary for arthroplasty surgeons who routinely manage PJI. Many risk factors have been identified in previous studies for PJI and two-stage exchange arthroplasty failure. These include patient-specific factors, such as end-stage renal disease (Deegan et al., 2014), obesity (Lok-Chi Man et al., 2020​​​​​​​), diabetes mellitus (Zmistowski and Alijanipour, 2013), and previously failed two-stage exchange (Kheir et al., 2017). A draining sinus tract represents an ultimate compromise of overlying soft tissues due to a chronic infectious process. Thus we hypothesized it would be a significant risk factor for treatment failure as well as failure to complete two-stage exchange arthroplasty.

Recently, per the 2018 International Consensus Meeting (ICM) on Musculoskeletal Infection, a draining sinus is now only considered a relative contraindication to one-stage exchange arthroplasty when the sinus cannot be excised or when the soft tissue defect is too large to reconstruct (Bialecki et al., 2019). However, our data suggest that the presence of a draining sinus tract may not be as benign as these recent recommendations suggest. In our study, a draining sinus tract was demonstrated to be a statistically significant risk factor in failing to undergo re-implantation. These data are consistent with previous studies investigating the negative outcomes associated with the presence of a sinus tract (Kandel et al., 2019; Xu et al., 2019). Further large-scale, prospective studies should be performed to refute or support these ICM recommendations and provide surgeons better insight into how to manage patients with a draining sinus tract.

Currently, no single investigation has reliably determined criteria for the successful eradication of infection after resection arthroplasty in two-stage exchange arthroplasty for PJI. Because of this, criteria for re-implantation differ by institution and even by surgeon. The criteria we use at our institution are as follows: (1) the patient has completed at least 6 weeks of antibiotics, (2) has normalized inflammatory lab values (WBC, ESR, CRP), and (3) has normalized nutrition labs (prealbumin, albumin, total protein). Our data suggest that a patient with a draining sinus is significantly less likely to meet these criteria for re-implantation. However, when accounting for all mechanisms of treatment failures including re-infection following re-implantation, there was no statistical difference detected between the two groups in terms of failure outcomes. This suggests that if a patient does meet criteria for re-implantation, it is still possible for them to perform similarly to those who had a draining sinus at presentation, illustrating the need for better defined criteria for re-implantation.

Mechanisms by which patients with a draining sinus fail to be re-implanted are likely multifactorial, and larger future studies are required to better elucidate the reasoning. Additionally, socioeconomic reasons could also preclude a patient receiving re-implantation. Examples of this include potential financial barriers to obtaining antibiotics as well as access and proper education regarding optimizing nutritional status. If the causative factors preventing re-implantation can be better identified, patients possessing these factors may have a better chance at being optimized prior to two-stage exchange arthroplasty or perhaps managed with an alternative treatment strategy.

This study has several important limitations that must be noted. Inherent limitations exist due to retrospective nature of this study, as well as the lack of MSIS guidelines prior to 2011. In addition, patient outcome scores were not considered in the definition of treatment success, and it is not reported whether patients in both cohorts had similar functional or quality of life after PJI. Lack of consistent medical documentation in regards to spacer antibiotic composition and soft tissue management is another limitation to this study. Additionally, due to the nature of this study, presence of a sinus tract is only shown to be associated with failure of re-implantation with causation being implied. Further conclusions from this data set are difficult to draw without the use of multiregression analysis, which is another limitation to this study. Finally, this study is limited by a relatively small sample size. However this sample size is similar to other studies investigating risk factors in prosthetic joint infection (Ford et al., 2018; Kheir et al., 2017).

## 5 Conclusions

In summary, the presence of a draining sinus appears to be significantly associated with failure to undergo re-implantation. Once explanted, it appears that patients with a sinus tract at presentation face greater barriers to re-implantation. Further studies investigating specific areas where patients fail to meet re-implantation requirements should be conducted to better elucidate the mechanisms by which this occurs. This study provides novel data upon which surgeons can use to better counsel their patients who present with a draining sinus and how that will affect their treatment algorithm for prosthetic joint infection.

## Data Availability

This study did not use a publicly available data set. Data were collected retrospectively from our own institution. Data can be made available upon request.
